# The complete chloroplast genome sequence of an alpine wildflower, *Philadelphus calvescens* (Rehd.) S. M. Hwang (Saxifragaceae)

**DOI:** 10.1080/23802359.2019.1693916

**Published:** 2019-12-09

**Authors:** Lijun Zhang, Dequan Zhang

**Affiliations:** a College of Pharmacy and Chemistry, Dali University, Dali, PR China;; b Institute of Materia Medica, Dali University, Dali, PR China

**Keywords:** Saxifragaceae, *Philadelphus calvescens*, complete chloroplast genome, phylogeny

## Abstract

*Philadelphus calvescens*, belonging to the family Saxifragaceae, is a beautiful shrub with white fragrant flowers which is widely distributed in southwest China. In this study, we sequenced the complete chloroplast (cp) genome of *P. calvescens* to investigate its phylogenetic relationship in Saxifragaceae. Total length of the cpDNA genome was 157,237 bp, consisting of a large single copy (LSC) region of 86,481 bp, a small single copy (SSC) region of 18,728 bp, and a pair of inverted repeat regions (IRs, 26,014 bp). The genome contained 114 genes, namely 79 protein-coding genes, 31 tRNA genes, and 4 rRNA. The overall GC content was 37.8%. Phylogenetic analysis suggested that *Philadelphus* was closely related to *Deutzia*. The present study was the first report on complete cp genome of *Philadelphus* so it could afford important genetic information for further researches on the genus and related genera.

*Philadelphus* L. is an important genus in Saxifragaceae, which includes approximately 70 species all over the world. Almost all of the species grow in the temperate regions of the Northern Hemisphere. There are 22 species in China and most of them possess important ornamental value (Pan et al. 2001). Most of the species in this genus are light-fast, drought-tolerant, cold-tolerant, and anti-caries (Liu et al. 2006). Due to these unique merits in physiological features, the species in this genus are ideal garden greening and honey source (Liu et al. 2011). *Philadelphus calvescens*, as a tall shrub growing in thickets, is widely distributed in northwestern Yunnan and southwestern Sichuan, China (Pan et al. 2001). It is usually used for cultivation and ornamental in the local areas. However, most of the studies on *Philadelphus* focused on cultivation and breeding of germplasm resources (Yu et al. 2014; Gao 2018), thus there was nearly no genetic report, as well as a genomic resource in the GenBank database to this day (22 September 2019). Therefore, we reported the complete chloroplast (cp) genome sequence of *P. calvescens* and explore its internal relationships within the family Saxifragaceae.

Total genomic DNA was extracted from fresh and clean leaves of *P. calvescens* sampled from Cangshan Mountains, Dali, Yunnan, China (N25°52′38.91″, E100°00′27.25″). At the same time, flowering branches of *P. calvescens* were collected which were used as voucher specimens, and deposited at the Herbarium of Medicinal Plants and Crude Drugs of the College of Pharmacy and Chemistry, Dali University (voucher number: ZDQ17030). Total DNA was extracted via modified cetyltrimethylammonium bromide (CTAB) method (Doyle 1987; Yang et al. 2014) and then sent to Novogene Company (http://www.novogene.com, China) for next-generation sequencing using Illumina Hiseq 2500 platform with pair-end (2 × 300 bp) library. The raw data were filtered using Trimmomatic version 0.32 with default settings (Bolger et al. 2014). Then paired-end reads of clean data were assembled into circular contigs using GetOrganelle.py (Jin et al. 2018) with *Deutzia crassifolia* (No. MG524993) as reference. Finally, the cpDNA was annotated using the online annotation tool the Dual Organellar Genome Annotato (DOGMA; http://dogma.ccbb.utexas.edu/) (Wyman et al. 2004) and tRNAscan-SE (Lowe and Chan 2016). Physical map of the cp genome was obtained via the web-based tool OGDraw version 1.2 (http://ogdraw.mpimp-golm.mpg.de/) (Lohse et al. 2013) and then the annotated cp genome sequences were submitted to GenBank (accession number: MN486873 and MN486874).

The *P. calvescens* cp genome is a double-stranded circular DNA of 157,237 bp in length. It exhibited a typical circular and quadripartite structure including two copies of inverted repeats (IRs, 26,014 bp), a large single copy (LSC, 86,481 bp), and a small single copy (SSC, 18,728 bp). A total of 114 genes were annotated, containing 79 protein-coding genes (PCGs), 31 transfer RNA genes (tRNA), and four ribosomal RNA genes (rRNA). Overall GC content was 37.8% and those in LSC, SSC, and IR regions were 35.9%, 31.8%, and 43.1%, respectively. Most of the genes did not contain the introns, whereas 16 contained one intron and two genes (*ycf3* and *clpP*) contained two introns.

A phylogenetic analysis was carried out using cp genome sequences of *P. calvescens* and other 12 cp genome sequences of species in Saxifragaceae that could be downloaded from the NCBI database. Meanwhile, two species from the Crassulaceae (*Sedum oryzifolium* and *S. sarmentosum*) were used as outgroups. After using MAFFT version 7.149 for aligning (Katoh and Standley 2013), jModelTest version 2.1.7 (Darriba et al. 2012) was used to determine the best-fitting model for these cp genomes. Finally, molecular phylogenetics of the species in Saxifragaceae was reconstructed based on the complete cp genomes using the Bayesian-Inference (BI) and Neighbor-Joining (NJ) methods. The BI analysis was conducted in MrBayes version 3.2.6 (Ronquist et al. 2012), and NJ phylogenetic tree was performed using MEGA7 (Kumar et al. 2016) with 1000 bootstrap replicates.

As a result, the two methods generated similar tree topology (Figure 1). The results showed that two individuals of *P. calvescens* could be clustered into an obvious lineage and *Philadelphus* is closely related to *Deutzia*, as well as *Hydrangea*. According to the reported genomes, *Hydrangea* and *Tiarella* were monophyletic groups. In total, this study reports the first complete cp genome of species in *Philadelphus* which would be beneficial to potential studies on phylogenetics of the genus and related group in Saxifragaceae.

**Figure 1.**
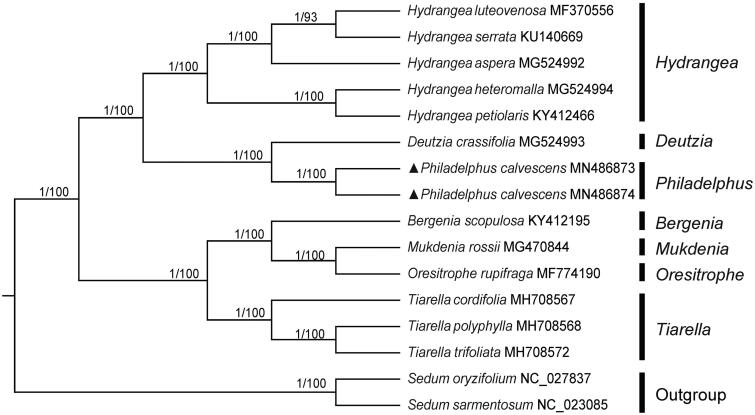
Phylogenetic tree of *Philadelphus* and related genus in Saxifragaceae based on the complete chloroplast genomes of 15 species, with *Sedum oryzifolium* and *S. sarmentosum* as outgroups. The numbers above each branch represent the posterior probabilities obtained from the BI analysis (before the slash) and the bootstrap values obtained from the NJ analysis (after the slash).
